# Geographic Differences in Lung Cancer Incidence: A Study of a Major Metropolitan Area within Southeastern Pennsylvania

**DOI:** 10.3390/ijerph17249498

**Published:** 2020-12-18

**Authors:** Yaqian Zhu, Thomas P. McKeon, Vicky Tam, Anil Vachani, Trevor M. Penning, Wei-Ting Hwang

**Affiliations:** 1 Department of Biostatistics, Epidemiology, and Informatics, Perelman School of Medicine, University of Pennsylvania, Philadelphia, PA 19104, USA; yazhu@pennmedicine.upenn.edu; 2 Center of Excellence in Environmental Toxicology, Perelman School of Medicine, University of Pennsylvania, Philadelphia, PA 19104, USA; mckeont@pennmedicine.upenn.edu (T.P.M.); avachani@pennmedicine.upenn.edu (A.V.); penning@upenn.edu (T.M.P.); 3 Department of Geography, Temple University, Philadelphia, PA 19122, USA; 4 Cartographic Modeling Laboratory, Perelman School of Medicine, University of Pennsylvania, Philadelphia, PA 19104, USA; vtam@pennmedicine.upenn.edu; 5 Abramson Cancer Center, Perelman School of Medicine, University of Pennsylvania, Philadelphia, PA 19104, USA; 6 Department of Medicine, Pulmonary, Allergy, and Critical Care Division, Hospital of University of Pennsylvania, Philadelphia, PA 19104, USA; 7 Departments of Systems Pharmacology and Translational Therapeutics, Perelman School of Medicine, University of Pennsylvania, Philadelphia, PA 19104, USA

**Keywords:** lung cancer, incidence, clustering, spatial autocorrelation, environmental risk factor

## Abstract

This study investigated the geographic variation and the clustering of lung cancer incidence rates in Philadelphia and the surrounding areas using addresses at the time of diagnosis. Using 60,844 cases from Pennsylvania Cancer Registry, we calculated and mapped the age-adjusted incidence rates for five Pennsylvania (PA) counties near Philadelphia between 1998–2007 and 2008–2017. We identified ZIP codes with significantly higher incidence rates than the state rates and examined their demographic and exposure characteristics. Further, we tested for spatial autocorrelation and identified spatial clusters using Moran’s I statistic. Our results showed that approximately one in four ZIP codes had an incidence rate that was significantly higher than the PA state rate in each period studied. Clusters of higher incidences were detected in the southeastern part of PA bordering New Jersey. These areas tended to be more populated, of lower socioeconomic status, and closer to manufacturing facilities and major highways. Possibly driven by the community and environmental factors, the observed differences in disease incidence suggest the importance of including residential location in risk assessment tools for lung cancer.

## 1. Introduction

Lung cancer is the leading cause of death from cancer in the United States, reporting 228,150 new diagnoses and 142,670 deaths in 2019 [1]. The national 5-year survival rate for 2010–2016 has been estimated to be 20.5% [1]. A recent report noted lung cancer constitutes the second most common type of cancer and the most common cause of cancer deaths in Pennsylvania (PA) in 2018 [2]. Although lung and bronchus cancer incidence rates in PA have been decreasing in recent years, Pennsylvania still consistently has a higher lung cancer incidence rate than the national average. The burden of lung cancer is even more prominent for major metropolitan areas such as Philadelphia and Pittsburgh. For example, the Centers for Disease Control and Prevention (CDC) State Cancer Profiles show that Pennsylvania’s 5-year age-adjusted incidence rate of 64.0 per 100,000 for 2014–2018 is higher than the national average of 59.2 per 100,000 while Philadelphia County’s reported incidence rate of 77.0 per 100,000 is statistically significant higher than the PA state rate [3].

Tobacco use through cigarette smoking has been established as a principal risk factor for lung cancer. However, 10%–20% of all lung cancer cases occur in never smokers, who are defined as people who smoked fewer than 100 cigarettes in their lifetime [4,5]. Environmental risk factors including exposure to air pollution, radon, asbestos, uranium, and diesel exhaust can also lead to lung cancer [6,7,8]. In fact, the International Agency for Research on Cancer (IARC) of the World Health Organization (WHO) has classified air pollution as a Group 1 carcinogen with clear evidence of being carcinogenic to humans, accounting for more than 230,000 lung cancer deaths per year worldwide [9,10]. These environmental exposures are likely to contribute to lung cancer development in never smokers and can increase lung cancer risk in smokers [11].

Because of the potential impact of environmental exposure on cancer risk, residential history should be included as a factor in population-based studies of lung cancer risk factors or risk assessment tools. Thus, it is critical to incorporate geographic location as an important component in identifying at-risk populations for cancer surveillance and screening beyond smoking history alone. Understanding geographic patterns in cancer incidence may be useful for devising public health interventions to optimize approaches to lung cancer screening and diagnosis. Furthermore, geographic analysis of lung cancer incidence may also enable us to gain insight into other cancer risk factors, such as proximity to industrial facilities that may produce toxic chemicals as well as other sources of air pollutants (e.g., traffic patterns).

The objective of this work is to examine the geographic variation in lung cancer incidence rates for Philadelphia and the surrounding counties to assess the impact of location on risk and disease burden. Using data from the Pennsylvania Cancer Registry, we employ statistical tools to identify areas with increased incidence risk for developing lung cancer and to detect hot spots of high lung cancer incidence. Further, we assess whether the population and environmental characteristics of these areas are associated with higher disease risk. The possible role that environment and community play in predisposing certain groups to lung cancer warrants its inclusion in lung cancer risk evaluations and promotes greater health surveillance in identified hot spots.

## 2. Materials and Methods

### 2.1. Study Area

**Figure 1:**
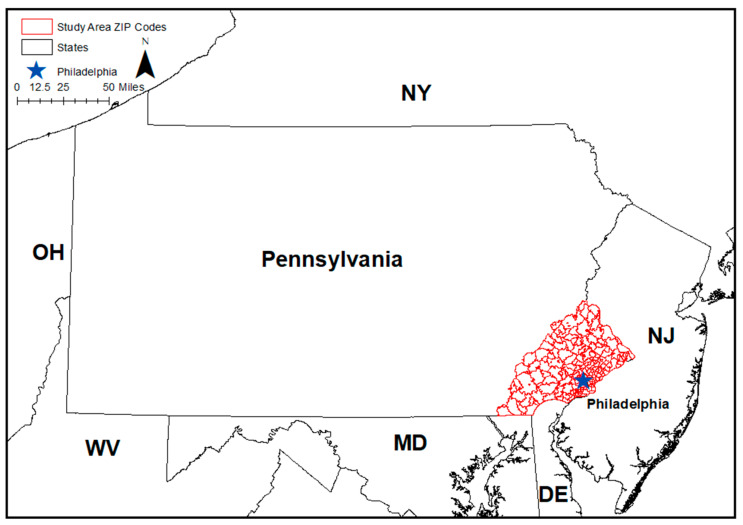
The study area in southeastern Pennsylvania. (NJ: New Jersey, DE: Delaware, MD: Maryland, WV: West Virginia, OH: Ohio, NY: New York).

The study area consisted of ZIP codes that fall into the five counties (Bucks, Chester, Delaware, Montgomery, and Philadelphia) in southeastern Pennsylvania. We chose to use ZIP code as the geographical unit of interest because of its familiarity in communications with health care providers and the general population. Even though ZIP codes typically have more variations within the unit than census tracts, many published reports have demonstrated findings from ZIP code level analyses to have similar utility to those that used census tracts [12,13,14]. Recent studies have used the ZIP code as their unit for analysis [15,16]. ZIP code boundaries for the year 2017 were used and sourced from the United States Census Bureau [17]. In total, 213 ZIP codes were included in the analysis. The study area of interest and its surrounding states are shown in Figure 1.

### 2.2. Data Sources

Case data were obtained from the Pennsylvania Cancer Registry (PCR), a statewide data system established in 1985 to collect records of all new cases of cancer diagnosed or treated in Pennsylvania. The PCR is an incidence-based registry, such that subsequent progression of diseases or relapses is not captured in the registry. The registry provides data on patient- and tumor-specific characteristics for individual cases and has earned Gold Certification from the North American Association of Central Cancer Registries (NAACCR), the highest level of data quality achieving at least 95% completeness, for all years of data available except 2001 (not certified) and 2012 (Silver Certification with at least 85% completeness) [18]. For this study, we restricted our analysis to (1) lung and bronchus cases using International Statistical Classification of Diseases and Related Health Problems, 10th revision (ICD 10) diagnosis codes for malignant neoplasm—C340 (main bronchus), C341 (upper lobe, bronchus or lung), C342 (middle lobe, bronchus or lung), C343 (lower lobe, bronchus or lung), C348 (overlapping sites of bronchus and lung), and C349 (unspecified part of bronchus or lung), (2) diagnoses between 1998 and 2017, the latest year which the data are available for research, and (3) those cases where residential address at the time of diagnosis was located within the above mentioned five counties in Pennsylvania. We conducted the present analysis under a data use agreement with Pennsylvania Department of Health and with the approval of the University of Pennsylvania Institutional Review Board (IRB number 831671).

To determine the geographical location at the time of diagnosis, the reported street addresses were geocoded using ArcGIS 10.6.1 software [19]. Only cases that matched on at least ZIP code level accuracy (65,261 cases, 99%) and addresses that were not a P.O. Box address (4362, 2%) were included in the subsequent analysis. A total of 60,899 lung and bronchus cancer cases met these criteria. Cases were further excluded if the matched ZIP codes no longer existed in the study area in 2015 (37, 0.06%) or if the age at diagnosis fell into an age group reported to have no population for the ZIP code of interest based on the government population estimates (see next section on Population size and covariate information), suggesting data entry error (18, 0.03%). The final sample size for analysis was 60,844 cases. We further classified each case into the major histology groupings (adenocarcinoma, squamous cell carcinoma, small cell carcinoma, large cell carcinoma, and other) using ICD-O-3 morphology codes and stages (local, regional, distant, other/unknown) using either 1977 or 2000 Surveillance, Epidemiology, and End Results (SEER) summary stage guidelines depending on the year of diagnosis [20,21,22]. Overall, 37.8% of the cases were adenocarcinoma, 18.8% were squamous cell carcinoma, and 13.2% and 10% were small cell and large cell carcinoma, respectively. The distribution for cases with local, regional, and distant stages was 32.6%, 17.2%, and 14.8%, respectively, and the remaining were unknown or un-staged.

### 2.3. Population Size and Covariate Information

**Figure 2:**
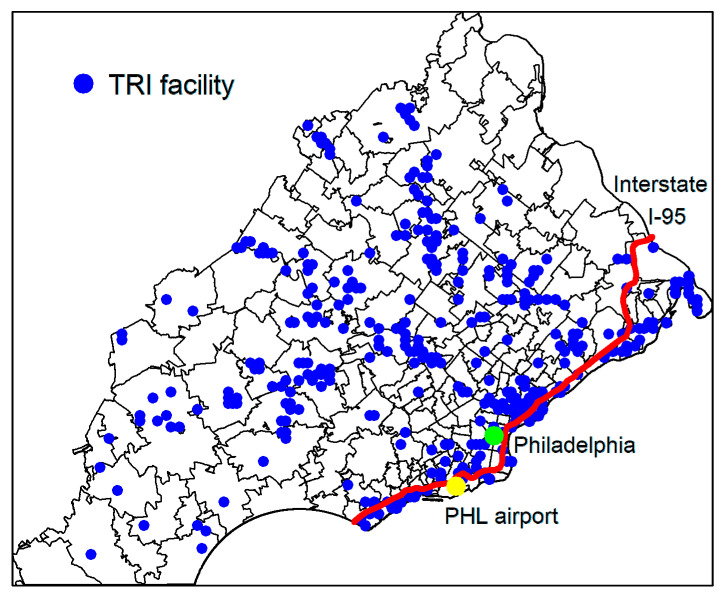
Locations of TRI facilities, Philadelphia (green point), Philadelphia International (PHL) Airport (yellow point), and Interstate I-95 highway (in red) in the study area.

The annual population size by age groups for a ZIP code was obtained from US Census Bureau using decennial census (2000, 2010), American Community Survey (ACS) 5-year estimates (2011 to 2017), or purchased through the data vendor Geolytics Inc. (Branchburg Township, NJ, USA) (2001 to 2009) [23,24]. Demographic information including median age, median income, percentage of whites, percentage of Hispanics, poverty level, percentage graduating high school or higher, employment to population ratio, and unemployment rates were obtained for each ZIP code using 2017 ACS estimates. Information on facilities reported to U.S. Environmental Protection Agency (EPA)’s Toxic Release Inventory (TRI) program [25], which tracks the management of toxic chemicals that may pose a threat to human health and the environment, were gathered from EPA’s Data Mart [26]. The total number of unique TRI facilities, total amounts of air emission as well as emissions from benzene, beryllium, cadmium, chloromethyl methyl ether, chromium compounds, crocidolite asbestos, and nickel compounds that fall within a 10-mile radius and a 15-mile radius of the centroid of a ZIP code between 1987 and 2017, and the distances to the nearest TRI facility from the centroid were computed [27]. These chemicals are chosen because they have been classified as Group 1 carcinogens affecting humans by IARC and as lung cancer-specific carcinogens according to the review by Cogliano et al. [9,28]. The shortest distances from each ZIP code’s centroid to Philadelphia International Airport (PHL) and Interstate-95 highway were computed using ArcGIS software. The locations of TRI facilities, the city of Philadelphia, PHL, and Interstate-95 highway are shown in Figure 2.

To explore whether the geographical patterns we observed in incidence rate can be explained by smoking, a key risk factor for lung cancer, we obtained the ZIP code level annual household expenditure on tobacco and smoking supplies provided by Geolytics as a proxy for cigarette use. The cigarette expenditure data are part of the Consumer Expenditure Survey (CES) collected by the US Bureau of Labor Statistics [29,30]. We computed the 5-year averaged annual expenditures per household using data from 2004 to 2008. Although it is not the goal of the current analyses to make inferences on association or causality, we have selected these years as an attempt to address the temporal relationship between smoking or cigarette use and lung cancer incidence.

### 2.4. Calculation of Age-Adjusted Incidence Rates

Because cancer tends to affect older people more and different ZIP codes may have different proportions of older individuals, we calculated age-adjusted incidence rates such that crude incidences for each ZIP code were externally adjusted according to the 2000 U.S. Standard Million Population, a commonly used standard population for adjustment that assumed a total population of 1,000,000 [3]. We specified 13 age groups distribution (0–4, 5–9, 10–14,15–19, 20–24, 25–34, 35–44, 45–54, 55–59, 60–64,65–74,75–84, 85 and above) as used by the ACS population estimates report. The age-adjusted incidence rate (reported as the number of cases per 100,000) for a ZIP code was calculated as $100,000\underset{k}{\sum }\frac{{\mathrm{cases}}_{k}}{{\mathrm{population}}_{k}}\cdot \frac{{\mathrm{std}.\mathrm{pop}}_{k}}{1,000,000}$, where $\frac{{\mathrm{cases}}_{k}}{{\mathrm{population}}_{k}}$ is the crude incidence rate and $std.pop_{k}$ is the standard population, respectively, for kth age group (k=1, …, 13). We calculated ten-year age-adjusted rates for the two time periods: 1998–2007 and 2008–2017. For each interval, the total number of cases and population size within the time period were summed over the years. We used 10-year intervals for simplicity and based on the reasonable assumption that incidence does not change substantially over consecutive years. Considering two time periods not only provided information on the general trend in lung cancer incidence in the past two decades but could also highlight important changes in the observed rates.

### 2.5. Mapping of Incidence Rates

The changes in the age-adjusted incidence rate from the first time period to the next were tested using Wilcoxon signed rank test. We calculated the percentage change in rates, and ZIP codes with rates higher in the 2008 to 2017 period than in the 1998 to 2007 period were identified. We then compared the rates against the five-year PA state age-adjusted rate for 2000–2004 and 2010–2014, which roughly correspond to the mid-point of each 10-year study time period, respectively. The state age-adjusted rates were obtained from the PA Department of Health’s Enterprise Data Dissemination Informatics Exchange (EDDIE) website [31], and the rates were 69.2 (95% CI: 68.6, 69.8) and 64.6 (95% CI: 64.1, 65.2) per 100,000 for 2000–2004 and 2010–2014, respectively. We determined whether the incidence rates were statistically higher than the state rate by computing 95% confidence intervals for the rate difference according to the method proposed by Tiwari et al. [32]. A 95% confidence interval that is completely above 0 would imply a statistically significant higher incidence rate than the state rate; ZIP codes with age-adjusted incidence rates in 2008–2017 that are significantly higher than the state rate were identified as “high incidence” ZIP codes. Additionally, rate ratios were calculated using the individual ZIP code rates and state rates. We reported the median and interquartile range (IQR) values of demographic and environmental characteristics and household expenditures on tobacco and smoking supplies for the high incidence ZIP codes versus remaining ZIP codes and compared them using Wilcoxon rank sum tests. Maps of 10–year age-adjusted incidence rates, the percentage change in rates, rate ratios, high incidence ZIP codes, and ZIP code characteristics were constructed using the *sp* package in R version 3.6.

### 2.6. Spatial Autocorrelation and Clustering

We assessed spatial autocorrelation and clustering using the Moran’s I statistic [33], the corresponding global test, and its Local Indicators of Spatial Association (LISA) [34]. Analogous to a correlation coefficient, the global Moran’s I statistic measures overall spatial autocorrelation of a quantity (e.g., incidence rate) by using the form of a correlation coefficient from every pair of the spatial units (e.g., ZIP codes) but weighted according to the spatial relationships among the units. The weights (also known as spatial weights) are used to represent neighboring relationships for each ZIP code under study in which two units that are closer in space would weigh more than the units that are farther away. We used the “queen” criterion to define spatial weights such that two ZIP codes were neighbors if they either share a common edge or share a common vertex and, thus, were more inclusive [35,36]. The difference in the results was minimal when the “rook” criterion was used, which defines another ZIP code as a neighbor only if they share a common edge.

A positive Moran’s I value would occur when incidence rates for neighbors are both greater or both less than the mean rate, and a negative value if the rate of a ZIP code is less than the mean and the rate for its neighbor is greater than the mean. A study region in which many high incidence ZIP codes neighbor other ZIP codes with high incidence would result in a large and positive I value. The global test for Moran’s I evaluated the overall patterns for the entire study region—whether the expressed trends in incidence were clustered (i.e., I > 0), dispersed (i.e., I < 0), or random (i.e., I ~ 0). The null hypothesis was that the incidence rates are randomly distributed among ZIP codes; that is, spatial processes underlying observed patterns were due to random chance [35,37]. We computed the associated *p*-value with a permutation test, which employed a conditional randomization assumption. To identify local spatial clusters (i.e., hot spots) in which ZIP codes with higher incidence rates tend to neighbor each other (i.e., high-high clusters), we considered LISA. These local indicators decomposed the global Moran’s I into individual contributions from each ZIP code and assessed the influence of individual spatial units on the magnitude of the global I value [34]. The LISA for each ZIP code indicated the extent of spatial clustering of similar incidence around that ZIP code and, thus, measured autocorrelation in smaller sub-regions [34].

We analyzed each 10-year time period separately and examined the pattern of high-high clusters across the two time periods. The global Moran’s I was calculated using the *moran.mc* function using 999 permutations, and local Moran’s I indicators were calculated using the function *localmoran* with the critical value set at 1.96 (or *p*-value < 0.05) for statistical significance. These functions may be found in the *spdep* package in R.

## 3. Results

### 3.1. Mapping of Age-Adjusted Lung Cancer Incidence Rates

**Figure 3:**
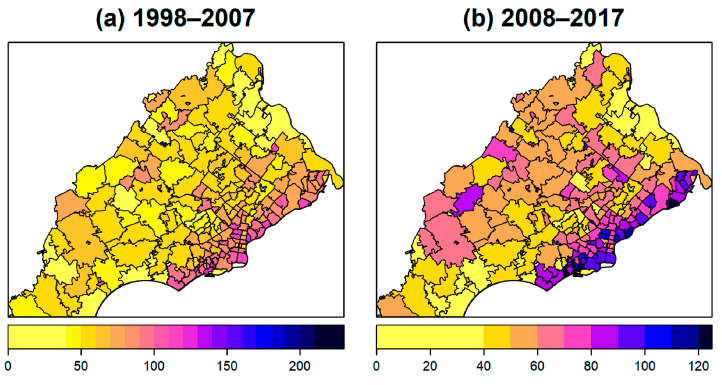
Maps of ten-year age-adjusted incidence rates per 100,000 for (**a**) 1998 to 2007 period, and (**b**) 2008 to 2017 period.

Maps of the age-adjusted lung cancer incidence rates for 1998–2007 and 2008–2017 are displayed in Figure 3. Across the 213 ZIP codes considered, the rates for 1998 to 2007 ranged from 0 to 292.96 and there were on average 69.72 lung cancer cases for every 100,000 people per year. The median was 66.37 and the first and third quartiles were 49.94 and 84.13, respectively, per 100,000. The rates for 2008 to 2017 ranged from 0 to 124.53 with a mean of 62.12 per 100,000; the median was 60.40 with the first and third quartiles of 46.91 and 77.88. One ZIP code was too small (e.g., <30) to provide a valid estimation of the incidence rate. ZIP codes with a higher incidence of lung cancer tended to lie along the lower right side of the study region, along the PA border with NJ. As shown in Supplementary Figures S1 and S2, the percentages of the major histology groups and stages in each ZIP code varied but no clear geographical patterns were observed.

**Figure 4:**
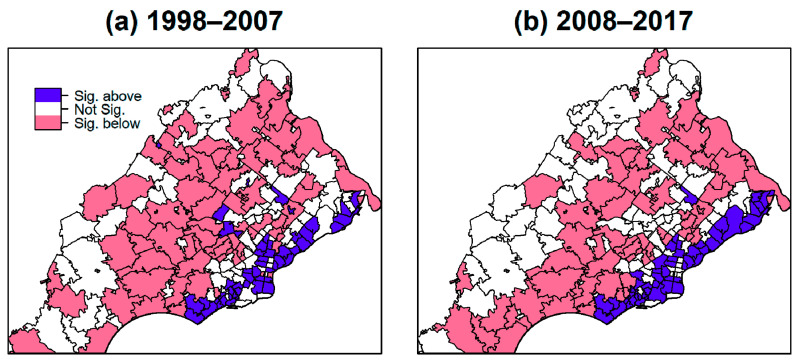
ZIP codes with age-adjusted incidence rates significantly higher than the age-adjusted PA state rates for (**a**) 1998 to 2007 period, and (**b**) 2008 to 2017 period.

There were 51 ZIP codes, accounting for 24% of the ZIP codes in each time period, that were significantly higher than the age-adjusted PA state rates, as shown in Figure 4; the two time periods shared 41 ZIP codes in common, suggesting these consistently had higher incidences of lung cancer. The ZIP codes with higher relative age-adjusted incidence rates tended to lie along the southeast part of the study area. Among cases from those ZIP codes, 35.4% were adenocarcinoma, 21.2% were squamous cell carcinoma, and 13.7% and 10.3% were small cell and large cell carcinoma, respectively. 33.1%, 16.9%, and 14%, were attributed to distant, regional, and localized stages, respectively, the numbers for which are similar to the distribution observed for the whole group.

**Figure 5:**
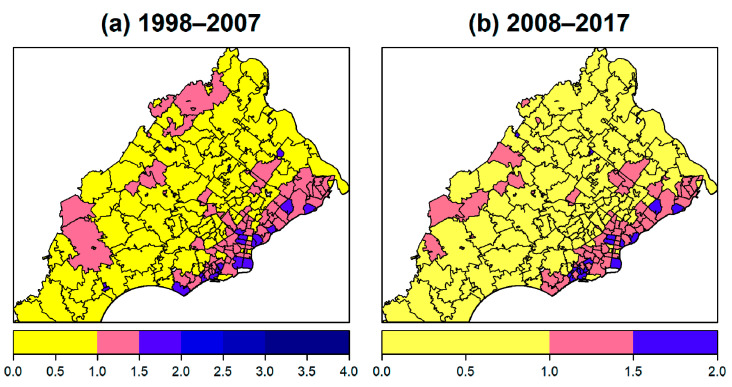
Maps of incidence rate ratio of each ZIP code’s rate to the PA state rate for (**a**) 1998 to 2007 period, and (**b**) 2008 to 2017 period.

The rate ratios of age-adjusted incidence rate relative to the age-adjusted incidence rate of PA state are shown in Figure 5. For 1998–2007, the mean rate ratio was 1.01, the median was 0.96, and the first and third quartiles were 0.72 and 1.22, respectively, while the maximum rate ratio was 4.23, indicating that some ZIP codes had rates that were over four times greater than the state rate. For 2008–2017, the mean rate ratio was 0.96, the median was 0.94, and the first and third quartiles were 0.74 and 1.21, respectively; the maximum ratio observed was 1.93, suggesting ZIP codes exist that had lung cancer rates that were almost double the state rate.

**Figure 6:**
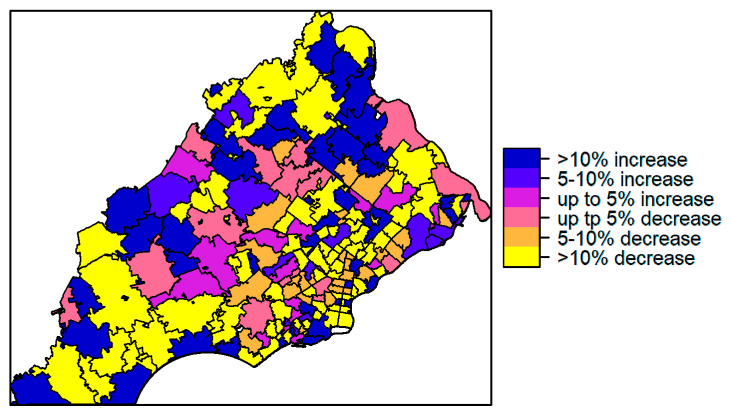
Maps of percentage changes in age-adjusted incidence rate from 1998–2007 period to 2008–2017 period.

We also observed a statistically significant difference in age-adjusted incidence rates between 1998–2007 and 2008–2017 (Wilcoxon signed rank test, *p* < 0.05). The median change in incidence rates from the first to the second ten-year periods was 0.074 with first and third quartiles of −0.060 and 0.172, respectively. The percent changes in rates are presented in Figure 6. Specifically, from 1998–2007 to 2008–2017, approximately 22% of the ZIP codes were observed to have over 10% increase in rates while 42% of ZIP codes had over 10% decrease in rates. 17% of ZIP codes had increased rates of over 15% while 29% had decreased rates of over 15% from the first to the second time period. There were no clear patterns in terms of which locations had an increase or decrease between the two time periods.

### 3.2. Characterizing ZIP Codes with High Incidence Rates

We observed significant differences between the 51 high incidence ZIP codes and the remaining ones with respect to all demographic variables examined (Wilcoxon rank sum tests, all *p*-values < 0.05). As shown in Table 1, ZIP codes with high incidence rates of lung cancer tended to have a larger population size and density, higher proportions of Hispanics, lower proportions of whites, and lower median age and were less wealthy as indicated by lower median incomes and higher poverty rates. Furthermore, residents in these ZIP codes also had significantly lower levels of education and higher unemployment rates.

Furthermore, high incidence ZIP codes were observed to have a significantly higher number of TRI facilities, tended to be located closer to these facilities, and had several times more air emissions recorded compared to the remaining ZIP codes (Wilcoxon rank sum tests, all *p* < 0.005). A closer look at individual compounds known to have carcinogenic effects indicated the same pattern of high incidence ZIP codes being exposed to a higher level of toxic emission. There were no significant differences in terms of distance to airport (*p* = 0.65), but high incidence ZIP codes were located significantly closer to I–95, a major interstate highway (*p* < 0.05). On the other hand, high incidence ZIP codes appeared to have a statistically significant (*p* < 0.05) lower median smoking spending (US$285.04) than the remaining ones (US$302.42). Maps showing the distribution of the demographic variables (Figure S3), TRI characteristics (Figure S4), and household expenditures on tobacco products (Figure S5) by ZIP code are provided in the Supplementary Materials.

### 3.3. Spatial Autocorrelation

**Figure 7:**
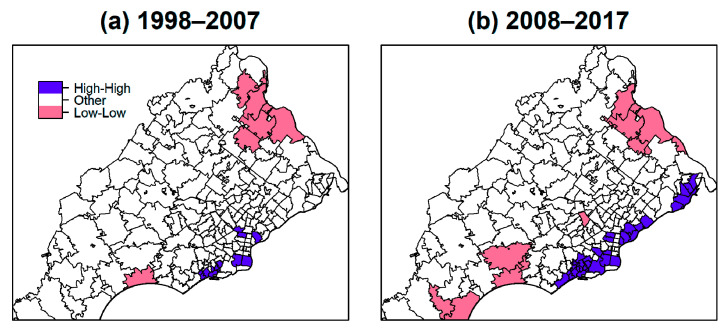
Local Indicators of Spatial Association (LISA) map with high-high and low-low incidence clusters for (**a**) 1998 to 2007 period, and (**b**) 2008 to 2017 period.

The global indices for Moran’s I were 0.136 for 1998–2007 and 0.411 for 2008–2017, indicating significant spatial autocorrelation (both *p* < 0.05). More specifically, ZIP codes with high incidence rates tended to be near others with high incidence and similarly for low incidence ZIP codes. Maps of LISA corresponding to Moran’s I for the two 10–year intervals are provided in Figure 7. Areas in red are clusters that had significant high-high autocorrelations for incidence rates (i.e., ZIP codes with high incidence neighbor other high incidence ZIP codes) included 12 ZIP codes for 1998–2007 and 33 ZIP codes for 2008–2017. Eleven of these (19,074, 19,094, 19,148, 19,022, 19,079, 19,029, 19,132, 19,134, 19,145, 19,032, 19,078) were in common to both sets and were among the 51 ZIP codes that were significantly higher than the state rates, indicating these ZIP codes consistently had greater lung cancer incidences. These high-high clusters were located along the southeast area near the border to New Jersey. This finding reinforced the observations from earlier analyses regarding the location of hotspots.

## 4. Discussion

In this study, we examined the geographic patterns of lung cancer incidence for Philadelphia and its surrounding regions over two ten-year periods from 1998 to 2017. We identified ZIP codes with high lung cancer incidence relative to the PA state rates and located clusters or “hotspots” of high incidences.

Our results demonstrated that lung cancer incidence rates were not the same across the ZIP codes in the five PA counties, suggesting disparity in environmental exposure or other risk factors. Almost all analyses suggested the southeastern study region inside Philadelphia county bordering New Jersey had higher rates of lung cancer. Local indicators of Moran’s I confirmed this general area as having clusters of significantly high incidence. These ZIP codes housed manufacturing industries in the past (some are remaining) and tracked closely along the I-95 highway. Factories and road traffic may be possible sources of hazardous exposures that can increase lung cancer risks. Although over half of the ZIP codes showed a decline in rates from 1998–2007 to 2008–2017, certain ones had high incidence across both time periods. Approximately one in four—making up 24% of those studied—ZIP codes in the study area had significantly higher incidence rates than that of the state as a whole in both time periods. Many of those ZIP codes were also identified to be in high incidence clusters with rate ratios ranging from 1.2 to 1.9.

Regions with increased rates may benefit from further investigation of the reasons for the observed trend. The high incidence ZIP codes were denser in terms of population size, poorer, and had lower percentages of whites and larger Hispanic populations. We further showed that these areas were in closer proximity to a greater number of TRI facilities, which released greater amounts of toxic emissions including many carcinogenic compounds, and to a major interstate highway. Surprisingly, given the established risk posed by smoking, the average household cigarette expenditure was lower for high incidence ZIP codes. Although cigarette expenditure is not a perfect measure of smoking behavior, this result may suggest that smoking may be more reflective of individual-specific risk; thus, associations between smoking and lung cancer may not transfer from the individual level to the group level (population in a certain zip code). When considering differences in risk between subpopulations, environmental factors may be more important. The comparison of high incidence ZIP codes to others pointed out additional factors to consider, emphasizing the contribution of one’s location. People who have known risk factors (e.g., smoking history, exposure to second-hand smoking, working in occupations that expose them to potential toxicants, etc.) may be more vulnerable and have the added burden of living in these areas. Characteristics and locations of these high incidence areas also matched with those for environmental justice communities [38], which are socially and economically disadvantaged and underserved. These findings suggested that identified communities may be disproportionately vulnerable to lung cancer and may require more attention.

### Limitations

There were some limitations in our study. Although lung cancer incidence rates are not expected to change substantially in consecutive years, we aggregated cancer cases into two ten-year periods that may have masked certain short-term fluctuations. The current study was also limited to one urban area and its surroundings. Although implementing this type of analysis to areas known to have high incidence provided more targeted and actionable impacts, our approaches are applicable for studying other geographical areas (e.g., state). We also did not have information on individual smoking history although we used cigarette expenditure as a proxy. However, it is unlikely smoking prevalence would be significantly varied sufficiently by ZIP code to fully explain the geographic variations observed. Furthermore, we only simplistically utilized TRI data in the current study and compared TRI data according to the subject’s place of residence at the time of diagnosis which may or may not be the same as the location of exposures. Although we observed greater overall air emissions and specific chemical exposures from ZIP codes with significantly higher incidence, the mechanisms by which these compounds contribute to lung cancer development remain unclear, nor did we consider differences in fugitive and stack air emissions which may affect dispersion. Determining the relative impact of each environmental toxicant and other known or unknown risk factors is beyond the scope of the current study, but future work should focus on conducting studies to confirm and estimate the magnitude of the associations observed in the current analyses.

## 5. Conclusions

Based on our geographic and clustering analyses of lung cancer incidence rates, we detected areas with high incidences in southeastern Pennsylvania along its border with New Jersey. These areas were associated with lower socioeconomic status and closer proximity to potential sources of pollution. Thus, environmental exposures and community risk factors corresponding to residential location may make certain individuals more susceptible to lung cancer, on top of the risk posed by smoking status. Future research is warranted to understand how risk factors identified in this study can be incorporated into existing risk assessment tools according to their relative effects on disease development and possible interactions between different factors. This may be accomplished by analyzing the significant risk factors we found in a spatial regression model. Public health professionals may use these tools to better identify individuals for targeted screening of lung cancer and greater surveillance.

## Figures and Tables

**Table 1:** Comparisons of demographic, Toxic Release Inventory (TRI), and distance characteristics: high incidence ZIP codes versus the remaining ZIP codes. Data are presented as median (IQR).

	Characteristics	High Incidence ZIP Codes (*n* = 51)	Remaining ZIP Codes (*n* = 161)	
Data Source		Median (IQR)	Median (IQR)	*p*-Value *
Demographic (2017 ACS)	Population size	21,685 (25,959)	12,473 (19,196)	0.00045
	Population density (/sq mi)	7216 (9788)	1548 (2607)	<0.00001
	Median age	36.9 (6.2)	42 (6.8)	<0.00001
	Median income (dollar)	52,859 (25,036)	88,558 (32,919)	<0.00001
	Percent White	72.7 (50.1)	87.8 (14.2)	<0.00001
	Percent Hispanic	5.2 (6.5)	3.3 (3.3)	0.000472
	Percent below poverty	11.6 (11.3)	3.1 (3.4)	<0.00001
	Percent with at least HS education	88.5 (8.4)	94.6 (5.5)	<0.00001
	Employment to pop ratio	59.0 (15.6)	64.3 (6.3)	0.000114
	Unemployment rate	7.8 (5.5)	5.0 (2.4)	<0.00001
TRI and distance (1987–2017 TRI)	No. of TRI facility/year ≤ 10 miles	148.5 (72.3)	78.0 (84.5)	<0.00001
	Total air emission ≤ 10 miles (ton)	23,898 (7162)	9120 (13,725)	<0.00001
	Benzene	1567.501 (1289.7)	10.197 (30.3)	<0.00001
	Beryllium	0 (0.3)	0 (0.3)	0.03630
	Cadmium	1.268 (4.7)	0.145 (2.7)	<0.00001
	Chloromethyl methyl ether	0 (0.1)	0 (0)	0.00007
	Chromium Compounds	154.949 (123.0)	15.129 (21.2)	<0.00001
	Crocidolite (Asbestos)	0 (0)	0 (0)	0.08244
	Nickel Compounds	160.575 (114.1)	9.721 (16.3)	<0.00001
	No. of TRI facility/year ≤ 15 miles	267.5 (68.5)	155.0 (186.5)	<0.00001
	Total air emission ≤ 15 miles (ton)	40,523 (12,550)	20,734 (26,047)	<0.00001
	Benzene	2147.298 (775.2)	18.101 (1363.4)	<0.00001
	Beryllium	0.26 (0)	0.26 (0.3)	0.02805
	Cadmium	5.560 (1.1)	2.750 (5.0)	<0.00001
	Chloromethyl methyl ether	0.103 (0)	0 (0.1)	<0.00001
	Chromium Compounds	173.379 (8.0)	35.056 (149.7)	<0.00001
	Crocidolite (Asbestos)	0 (0)	0 (0.8)	0.01102
	Nickel Compounds	192.075 (150.5)	22.500 (113.0)	<0.00001
	Nearest TRI facility (meters)	1161 (1248)	2677 (3417)	<0.00001
	Distance to PHL airport (meters)	22,451 (11,876)	23,020 (21,394)	0.6558
	Distance to I-95/major highway (meters)	3076 (3429)	18,811 (22,116)	<0.00001
Cigarette Use (2004–2008 CES)	Annual household expenditure for tobacco and smoking supplies (dollar)	285.04 (26.8)	302.42 (7.5)	<0.00001

*: *p*-values are based on Wilcoxon rank sum test.

## Supplementary Materials

The following are available online at https://www.mdpi.com/1660-4601/17/24/9498/s1: Maps of the characteristics analyzed in Table 1 are presented. Figure S1: Percentages of lung cancer cases by the major lung cancer histology types: (a) adenocarcinoma, (b) squamous cell, (c) small cell, and (d) large cell. Figure S2: Percentages of lung cancer cases by the SEER summary stages classification: (a) distant stage, (b) regional stage, (c) local stage. Figure S3: Demographic characteristics: (a) Population size, (b) population density, (c) median age in years, (d) median income in $10,000, (e) percentage of the population who are white, (f) percentage of the population who are Hispanic, (g) percentage below poverty line, (h) percentage with at least a high school education, (i) employment to population ratio, and (j) unemployment rate. Figure S4: Amount of air emission (in tons) within 10 miles of a ZIP code’s centroid for chemicals suggested to be possible lung carcinogens: (a) benzene, (b) beryllium, (c) cadmium, (d) chloromethyl methyl ether, (e) chromium compounds, (f) crocidolite (asbestos), and (g) nickel compounds. Figure S5: Median averaged annual household expenditures (2004–2008) on tobacco products or smoking supplies.
