# Supplementary material for: Geographic Differences in Lung Cancer Incidence: A Study of a Major Metropolitan Area within Southeastern Pennsylvania
Source: Int J Environ Res Public Health. 2020 Dec 18;17(24):9498. doi: 10.3390/ijerph17249498 (PMC7767044; doi:10.3390/ijerph17249498)
Supplement: Supplementary file 1 [file ijerph-17-09498-s001.pdf]

Supplementary Materials for

Geographic Differences in Lung Cancer Incidence: A Study of a Major Metropolitan Area within Southeastern Pennsylvania

Yaqian Zhu [1], Thomas P. McKeon [2,3], Vicky Tam [4] Anil Vachani [2,5,6], Trevor M. Penning [2,5,7], and Wei-Ting Hwang [1,2,5]

1. Department of Biostatistics, Epidemiology, and Informatics, Perelman School of Medicine, University of Pennsylvania, Philadelphia, PA 19104, USA
2. Center of Excellence in Environmental Toxicology, Perelman School of Medicine, University of Pennsylvania, Philadelphia, PA 19104, USA
3. Department of Geography, Temple University, Philadelphia, PA 19122, USA
4. Cartographic Modeling Laboratory, Perelman School of Medicine, University of Pennsylvania, Philadelphia, PA 19104, USA
5. Abramson Cancer Center, Perelman School of Medicine, University of Pennsylvania, Philadelphia, PA 19104, USA
6. Department of Medicine, Pulmonary, Allergy, and Critical Care Division, Hospital of University of Pennsylvania, Philadelphia, PA 19104, USA
7. Departments of Systems Pharmacology and Translational Therapeutics; Perelman School of Medicine, University of Pennsylvania, Philadelphia, PA 19104, USA

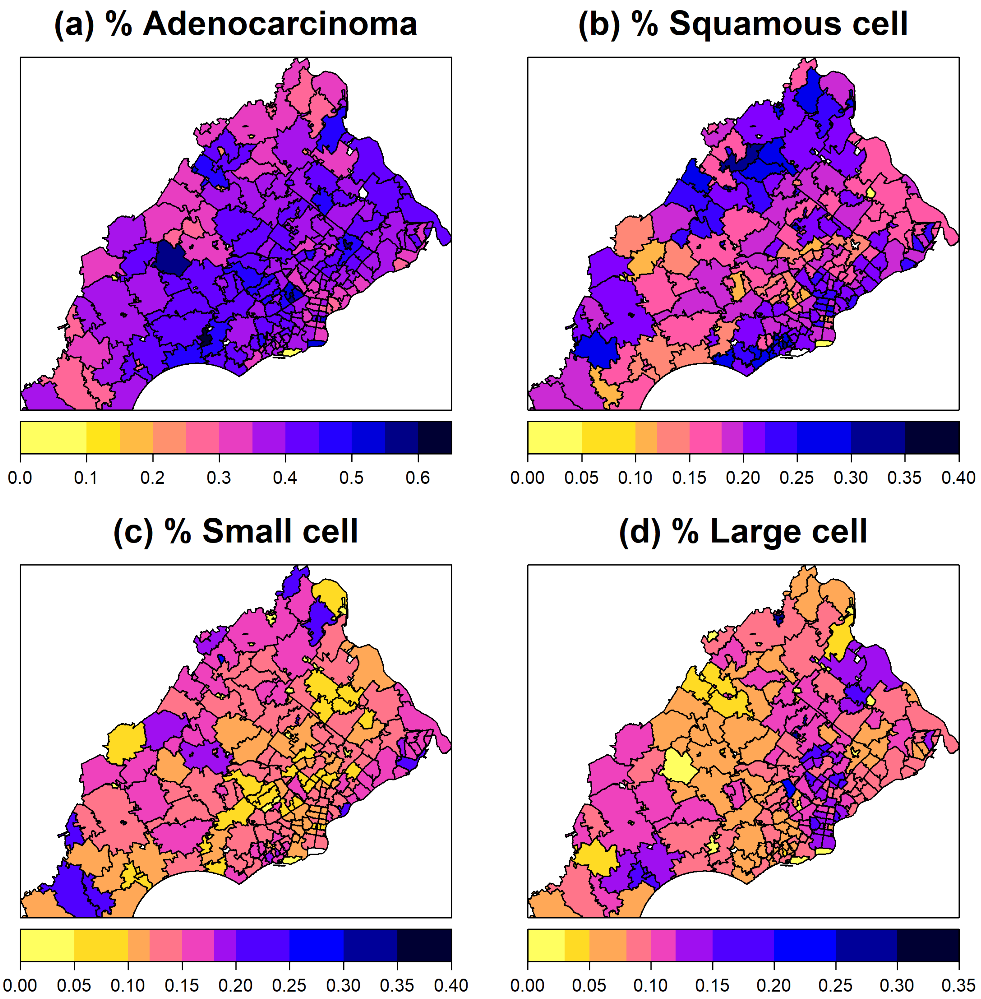

Figure S1: Percentages of lung cancer cases by the major lung cancer histology types: (a) adenocarcinoma, (b) squamous cell, (c) small cell, and (d) large cell.

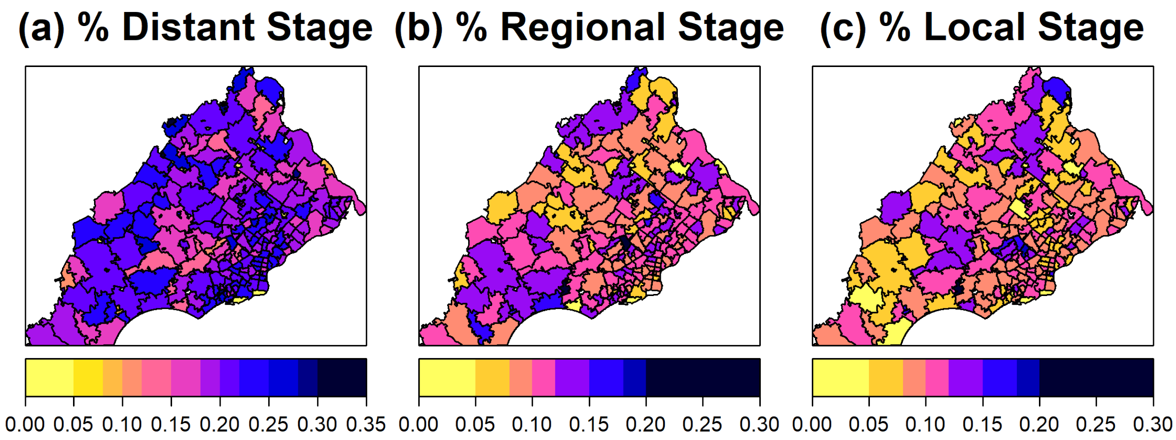

Figure S2: Percentages of lung cancer cases by the SEER summary stages classification: (a) distant stage, (b) regional stage, (c) local stage.

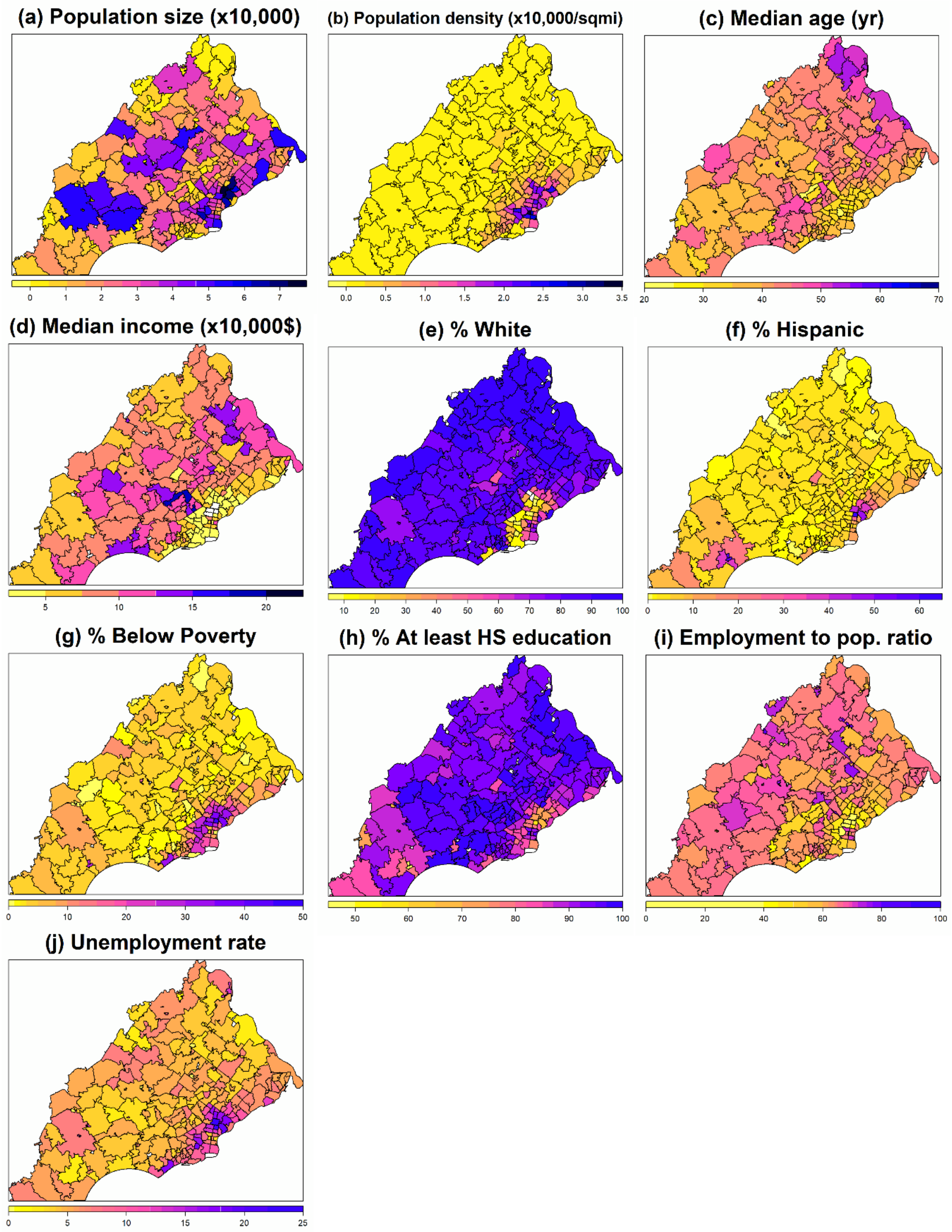

Figure S3: Demographic characteristics as shown in Table 1: (a) Population size, (b) population density, (c) median age in years, (d) median income in $10,000, (e) percentage of the population who are white, (f) percentage of the population who are Hispanic, (g) percentage below poverty line, (h) percentage with at least a high school education, (i) employment to population ratio, and (j) unemployment rate.

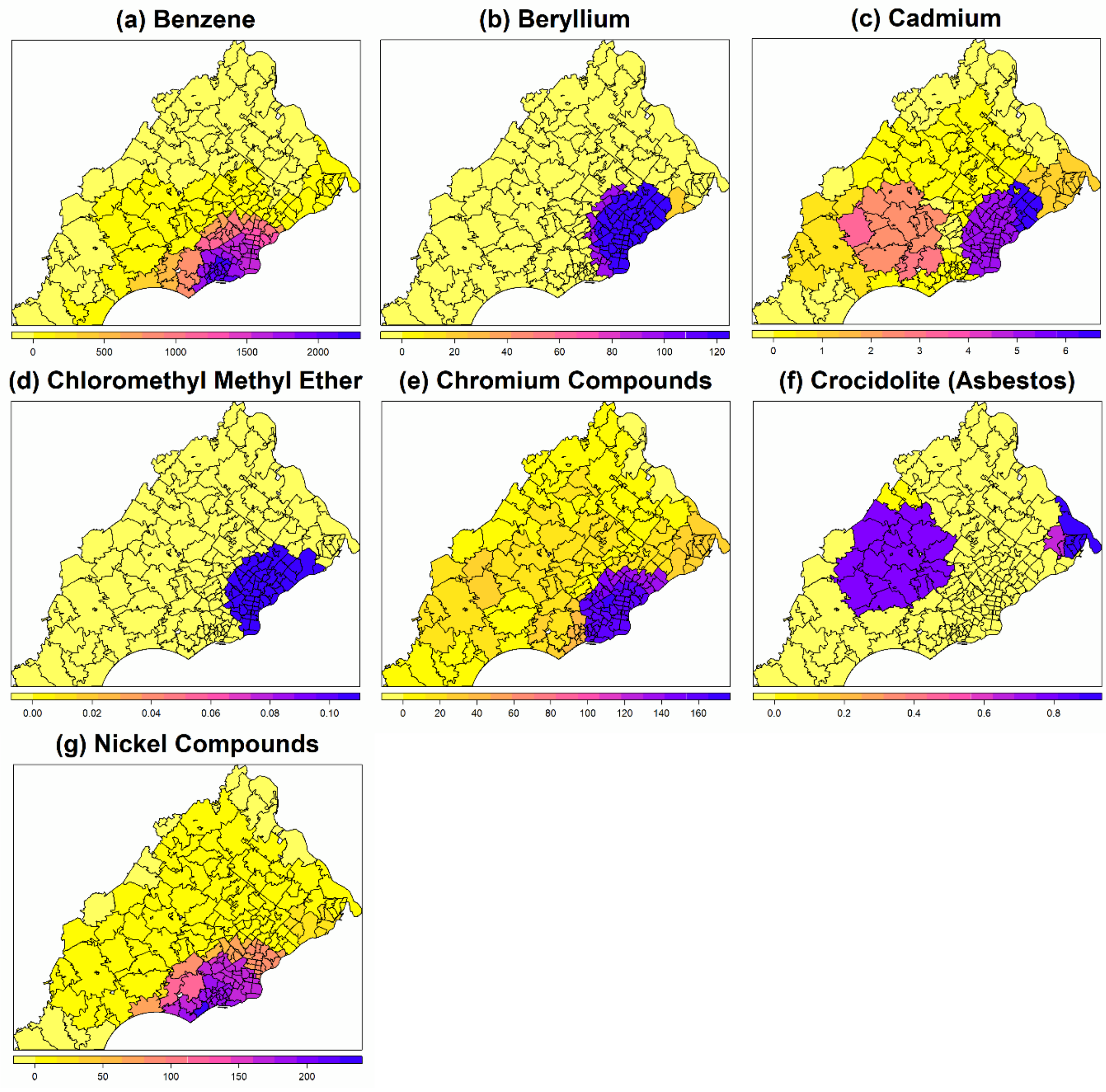

Figure S4: Amount of air emission (in tons) within 10 miles of a ZIP code's centroid for chemicals suggested to be possible lung carcinogens as shown in Table 1: (a) benzene, (b) beryllium, (c) cadmium, (d) chloromethyl methyl ether, (e) chromium compounds, (f) crocidolite (asbestos), and (g) nickel compounds.

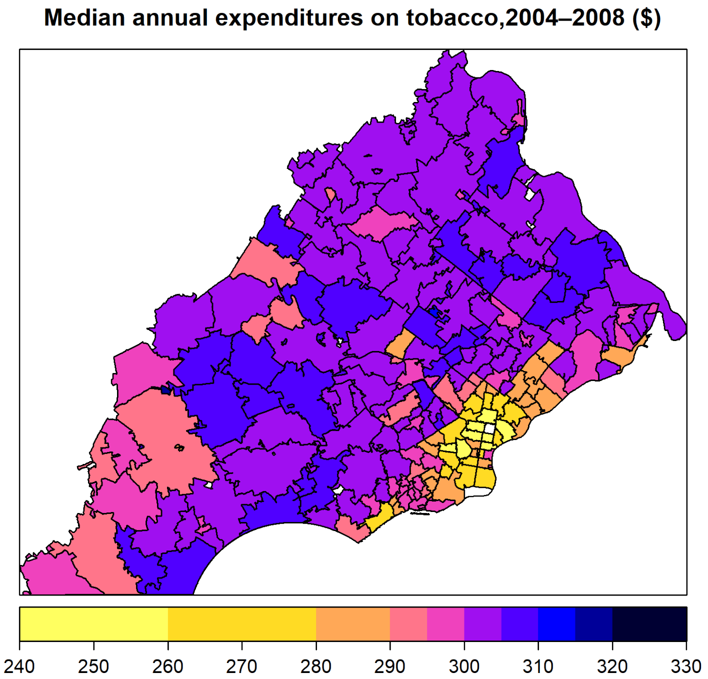

Figure S5: Median averaged annual household expenditures (2004–2008) on tobacco products or smoking supplies as shown in Table 1.
